# Serum Protein Profile at Remission Can Accurately Assess Therapeutic Outcomes and Survival for Serous Ovarian Cancer

**DOI:** 10.1371/journal.pone.0078393

**Published:** 2013-11-11

**Authors:** Jinhua Wang, Ashok Sharma, Sharad A. Ghamande, Stephen Bush, Daron Ferris, Wenbo Zhi, Mingfang He, Meiyao Wang, Xiaoxiao Wang, Eric Miller, Diane Hopkins, Michael Macfee, Ruili Guan, Jinhai Tang, Jin-Xiong She

**Affiliations:** 1 Center for Biotechnology and Genomic Medicine, Georgia Health Sciences University, Augusta, Georgia, United States of America; 2 Sino-American Cancer Research Institute at Nanjing University of Technology and Jiangsu Cancer Hospital, Nanjing, Jiangsu province, China; 3 Jiangsu Cancer Hospital, Nanjing Medical University, Nanjing, Jiangsu, China; 4 Institute of Translational Medicine, School of Pharmaceutical Sciences, Nanjing University of Technology, Nanjing, Jiangsu province, China; 5 Department of Obstetrics and Gynecology, Georgia Health Sciences University, Augusta, Georgia, United States of America; 6 Institute of Urological Surgery, First Hospital of Beijing University, Beijing, China; The University of Kansas Medical Center, United States of America

## Abstract

**Background:**

Biomarkers play critical roles in early detection, diagnosis and monitoring of therapeutic outcome and recurrence of cancer. Previous biomarker research on ovarian cancer (OC) has mostly focused on the discovery and validation of diagnostic biomarkers. The primary purpose of this study is to identify serum biomarkers for prognosis and therapeutic outcomes of ovarian cancer.

**Experimental Design:**

Forty serum proteins were analyzed in 70 serum samples from healthy controls (HC) and 101 serum samples from serous OC patients at three different disease phases: post diagnosis (PD), remission (RM) and recurrence (RC). The utility of serum proteins as OC biomarkers was evaluated using a variety of statistical methods including survival analysis.

**Results:**

Ten serum proteins (PDGF-AB/BB, PDGF-AA, CRP, sFas, CA125, SAA, sTNFRII, sIL-6R, IGFBP6 and MDC) have individually good area-under-the-curve (AUC) values (AUC = 0.69–0.86) and more than 10 three-marker combinations have excellent AUC values (0.91–0.93) in distinguishing active cancer samples (PD & RC) from HC. The mean serum protein levels for RM samples are usually intermediate between HC and OC patients with active cancer (PD & RC). Most importantly, five proteins (sICAM1, RANTES, sgp130, sTNFR-II and sVCAM1) measured at remission can classify, individually and in combination, serous OC patients into two subsets with significantly different overall survival (best HR = 17, p<10^−3^).

**Conclusion:**

We identified five serum proteins which, when measured at remission, can accurately predict the overall survival of serous OC patients, suggesting that they may be useful for monitoring the therapeutic outcomes for ovarian cancer.

## Introduction

Ovarian cancer (OC) is the fifth-leading cause of cancer death among woman in the United States, accounting for approximately 3% of all new cancer patients [1]. The American Cancer Society estimates that in 2013, about 22,240 new cases of ovarian cancer will be diagnosed and 14,030 women will die of ovarian cancer in the United States. Worldwide, this disease is the sixth most common cancer in women, causing 140,200 deaths in 2010 [2]. Unfortunately, most patients (∼70%) are diagnosed with advanced stages of the disease with poor prognosis. Although advances in chemotherapy and improved understanding of genetic risk factors and molecular pathogenesis have provided new treatment possibilities, the 5-year survival rates with optimal debulking surgery and intra-peritoneal chemotherapy are close to 50%. However, the rates of long-term survival (>10 years) in patients diagnosed with early-stage (stage I or II) are 80–95% [3]. The lack of successful treatment strategies led to a need for seeking novel approaches to detect this disease in early stage and treat this disease effectively in the advanced stage. Recently, there has been a surge of interest in exploring the genome and proteome for biomarkers that may aid in early detection, diagnosis and monitoring of therapeutic outcome and recurrence.

Previous biomarker research has mostly focused on the discovery and validation of diagnostic biomarkers, especially those that can detect OC at an early stage. The glycoprotein CA125 is the most widely used biomarker for ovarian cancer. It is elevated in approximately 80% of patients with advanced cancer; however, despite its high sensitivity, it lacks specificity and, therefore, has limited positive predictive value (PPV) for population screening, especially for early stage cancer. Extensive search for better biomarkers has been carried out in the last few years and has led to the discovery of a large number of potentially new OC biomarkers including the recently FDA-approved human epididymis protein 4 (HE4) [4], [5]. These new biomarkers individually do not perform better than CA125 but biomarker panels with or without CA125 generally perform better than CA125 or other individual biomarkers [6]–[10]. Although the currently available biomarkers do not yet have sufficient PPV suitable for population screening [11], diagnostic biomarker is a very active and rapidly advancing area of research in ovarian cancer [12].

Biomarkers that allow accurate assessment of therapeutic outcome may significantly improve patient care. After the initial cytoreductive surgery and combination chemotherapy, the majority of OC patients are believed to achieve a complete clinical remission [13]. In the remission stage, CA125 is routinely monitored during the follow-up and it is widely used as a biomarker for remission. Although CA125 is clearly reduced and returned to levels observed in controls, CA125 may not detect residual cancer cells. After therapy, the patients may have completely remitted or the tumor cell number and size becomes very small so that the residual tumor cannot be detected by tumor antigens such as CA125. However, as the tumor cells are still present within such patients in subclinical status, the immune system of the patients may be responding to the tumor cells. Therefore, inflammatory molecules may be abnormal in patients with subclinical phenotypes [14]–[16]. In this study, we profiled over 40 serum proteins including immune markers in sera from OC patients to develop biomarkers that may be useful to assess the therapeutic outcomes.

## Patients and Methods

### Human Subjects and Serum Samples

This study was approved by the institutional review board of the Georgia Regents University and written informed consent was obtained from every subject or a legally authorized representative. All the consents were filed properly in records and were also stored in searchable database. Consent procedure used in this study was approved by the ethics committee of Georgia Regents University. The subjects used in this study included 75 ovarian cancer patients and 70 healthy women as control. All the patients with ovarian cancer in this study were from an academic gynecologic oncology practice in Georgia, USA. Disease progression was defined by either CA125 levels ≥2 × nadir value on two separate occasions (GCIG criteria), or by an increase in measurable lesions as per RECIST criteria [17]. Patient’s conditions were staged according to the criteria of the International Federation of Gynecology and Obstetrics (FIGO). The age distribution and tumor characteristics of the patient population are presented in Table 1. Only high-grade (serous) cancers were included to have homogenous samples and low-grade cancers (mucinous and clear cell) were dropped from this study. A total of 101 serum samples from 75 patients were obtained at 3 different stages of disease progression: post-diagnosis (PD, n = 25), recurrent (RC, n = 43) and remission (RM, n = 33).

**Table 1:** Characteristics of the patient population.

		Control (n = 70)	PD (n = 25)	RC (n = 43)	RM (n = 33)	p-value
**Age(year)**						
	Mean ± SD	59.97±6.82	65.32±11.27	63.96±11.26	61.10±12.04	ANOVA
	Median	59.96	66.38	64.53	61.56	p = 0.06
	Range	(36.62–80.33)	(37.52–87.24)	(39.10–88.96)	(27.48–83.12)	
**FIGO staging**						
	Stage I		1	4	10	Chi-squared
	Stage II		1	4	3	p = 0.09
	Stage III		21	32	19	
	Stage IV		2	3	1	
**Histological type**						
	Serous		25	43	33	
**Tumor grade**						
	Grade 1		0	5	7	Chi-squared
	Grade 2		3	9	4	p = 0.10
	Grade 3		22	29	22	
**Surgery type**						
	optimal		14	27	24	Chi-squared
	suboptimal		11	16	9	p = 0.40

A total of 101 serum samples from 75 patients were obtained at 3 different stages of disease progression: post-diagnosis (PD, n = 25), recurrent (RC, n = 43) and remission (RM, n = 33).

### Luminex Assays

The Luminex kits were obtained from Millipore (Billerica, MA, USA) and assays were performed as per manufacturer’s instructions to determine the serum levels of 40 molecules. Properly diluted serum samples were incubated with the antibody-coupled microspheres and then with biotinylated detection antibody before the addition of streptavidin-phycoerythrin. The captured bead-complexes were measured with FLEXMAP 3D system (Luminex Corporation, Austin, TX, USA).

### Statistical Analyses

All statistical analyses were performed using the R language and environment for statistical computing (R version 2.12.1; R Foundation for Statistical Computing; www.r-project.org). We used both single protein and multi-marker models for the classification of cases and controls. Only 4 to 8 best performing proteins were selected for multi-marker models and linear discriminate analysis was performed using combinations of 3 proteins. The performance of each model was evaluated using the leave-one-out cross validation method. The area-under-the-curve (AUC) of the receiver-operating-characteristic (ROC) curves was computed to perform comparison of different models. We used Cox proportional hazards models to evaluate the impact of serum protein levels on survival. Overall survival was calculated as time from diagnosis date to the death of patient. Patients who are alive with no evidence of disease were censored at the date of last follow-up visit. Univariate analyses were performed by using the Kaplan-Meier plots, and statistical significance between survival curves was assessed using the log rank test. To assess the combined effect of different proteins on survival, multivariate analysis was performed using proteins having significant effect in the univariate analysis.

## Results

### Twenty-five Serum Proteins are Altered in Serous OC

**Figure 1:**
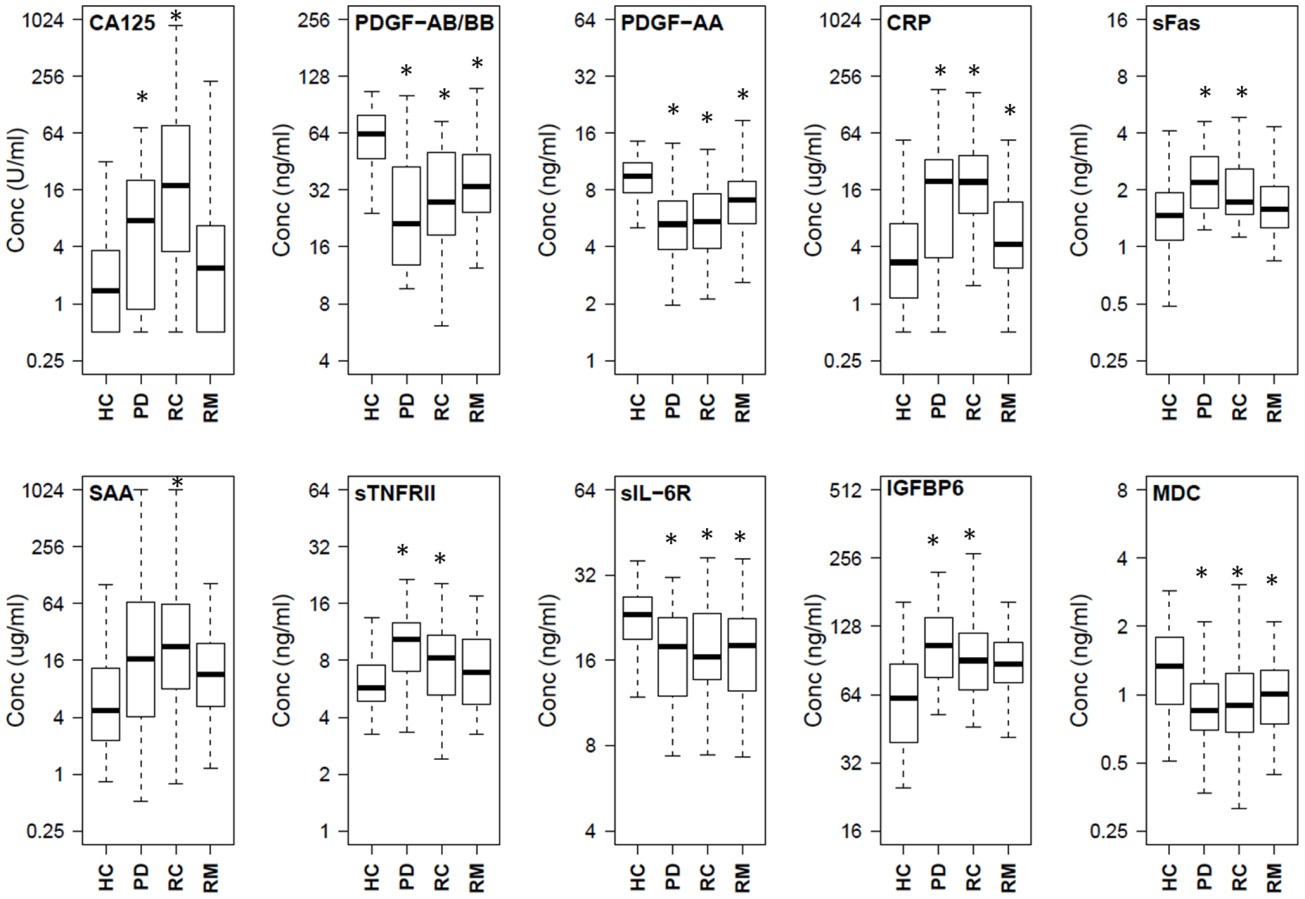
Boxplots representing the serum protein levels in patient subgroups and healthy controls. PD: Post Diagnosis, RC: Recurrence, RM: Remission, HC: Healthy Controls. (*) significant difference as compared to healthy controls.

Serum levels of the 40 proteins in the PD, RC and RM groups were compared with healthy controls (HC) using a student’s t-test. Significant differences were found for 25 proteins in at least one of the three groups as compared to the HC group (Table 2). Box plots for ten representative proteins are shown in Figure 1. The data for all 40 proteins is provided in Table S1 in File S1.

**Table 2:** Significant changes in serum protein levels in patients as compared to healthy controls (PD: Post Diagnosis, HC: Healthy Controls, RC: Recurrence, RM: Remission).

Protein	PD/HC	p-val*	RC/HC	p-val*	RM/HC	p-val*
**CA125**	**3.46**	**0.011**	**11.40**	**2E-07**	1.56	0.249
**CRP**	**3.52**	**0.037**	**6.35**	**2E-07**	**1.95**	**0.045**
**PDGF-AB/BB**	**0.39**	**1E-05**	**0.43**	**5E-07**	**0.52**	**2E-04**
**PDGF-AA**	**0.60**	**4E-04**	**0.56**	**1E-06**	**0.72**	**0.005**
**sCD40L**	**2.48**	**1E-04**	**2.26**	**7E-04**	**2.27**	**3E-04**
**IGFBP-2**	**4.69**	**2E-04**	1.63	**0.279**	1.27	0.603
**sFas**	**1.57**	**2E-04**	**1.33**	**0.006**	1.13	0.322
**sIL-6R**	**0.74**	0.008	**0.74**	**2E-04**	**0.75**	**0.002**
**SAA**	2.81	0.080	**4.11**	**3E-04**	1.57	0.230
**Leptin**	1.13	0.796	**2.91**	**4E-04**	**2.79**	**0.001**
**sVCAM-1**	0.86	0.185	**0.75**	**7E-04**	0.84	0.052
**MDC**	**0.69**	**0.004**	**0.72**	**0.020**	**0.79**	**0.042**
**sIL-4R**	0.90	0.549	**0.68**	**0.004**	0.82	0.220
**sE-SELECTIN**	**0.66**	**0.011**	**0.71**	**0.006**	**0.71**	**0.039**
**tPAI-1**	**0.73**	**0.018**	**0.74**	**0.011**	**0.73**	**0.006**
**CD14**	**1.29**	**0.006**	**1.22**	**0.042**	1.11	0.309
**MMP-1**	1.67	0.097	**1.98**	**0.008**	1.21	0.490
**sTNFRII**	**1.45**	**0.015**	**1.31**	**1E-02**	1.17	0.180
**IGFBP-6**	**1.56**	**0.015**	1.40	**0.048**	1.35	0.093
**HGF**	1.21	0.263	1.31	**0.018**	1.19	0.309
**sICAM-1**	0.87	0.309	0.86	0.222	**0.78**	**0.024**
**IGFBP-1**	**1.81**	**0.025**	0.99	0.974	1.20	0.546
**sIL-2Ra**	**1.38**	**0.038**	1.08	0.616	0.97	0.853
**MMP-9**	1.39	0.309	1.55	0.053	**1.55**	**0.045**
**CA15-3**	1.33	0.263	**1.56**	**0.049**	1.17	0.501

* The p-values are adjusted for multiple testing using FDR method.

### Protein Panels Accurately Distinguish Active Cancer from Controls

**Figure 2:**
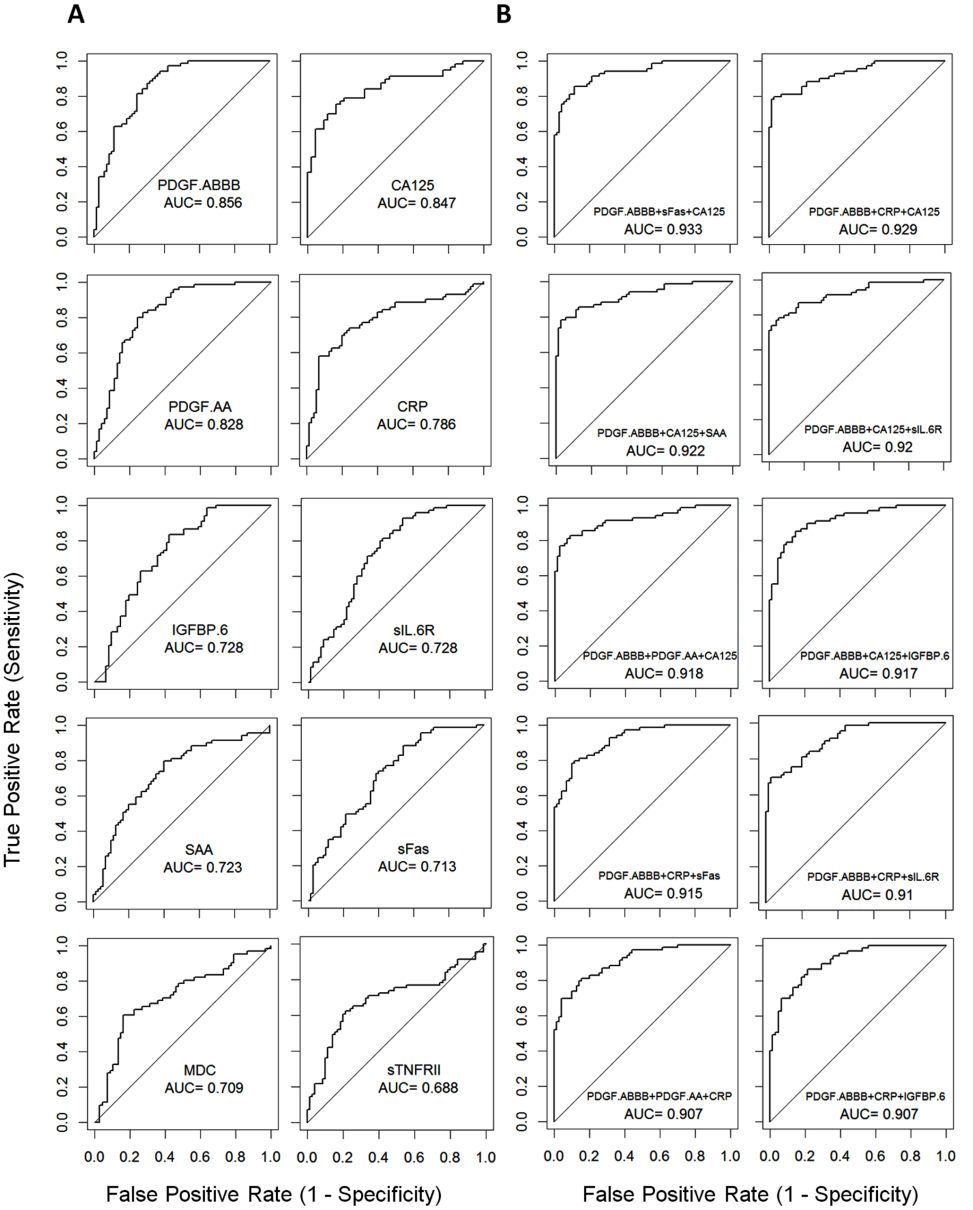
The ROC curves for the top molecules that can distinguish cancer patients (post diagnosis and recurrence) from healthy controls. Single proteins (A) and multi-marker models (B) were used for the classification analyses. For multi-marker models, linear discriminate analysis was performed using combinations of 3 proteins. The diagnostic performance of each model was evaluated using leave one out cross validation method. The utility of serum proteins as ovarian cancer biomarkers was evaluated using the area-under-curve (AUC) of the ROC curves for different models.

The utility of serum proteins as OC biomarkers was initially evaluated using AUC values. The top 10 molecules that can distinguish cancer (PD + RC) from HC are shown in Figure 2A. The three best performing molecules are PDGF-AB/BB (AUC = 0.856), CA125 (AUC = 0.847) and PDGF-AA (AUC = 0.828). CRP, IGFBP6 and sIL6R also have excellent AUC (0.786, 0.728 and 0.728, respectively). It is well known that combinations of molecules may significantly improve the performance of biomarkers. We searched for 3-protein models by using the top 8 proteins selected based on the single protein AUC values (resulting in a total of 56 models) (Table S3 in File S1). The AUC of the individual proteins was in the range of 0.688 to 0.856 (Figure 2A). The top ten three-marker models are illustrated in Figure 2B. The best model (PDGF − AB/BB + CA125 + sFas) has an AUC value of 0.933 and 12 models have AUC values greater than 0.90, significantly better than the two best individual proteins (AUC = 0.856 for PDGF-AB/BB and AUC = 0.847 for CA125). It is not surprising that all ten top models contain PDGF-AB/BB as one of the three proteins.

### Serum Profile at Remission is Distinct from Both Active Cancer and Controls

Eleven proteins were significantly different between RM and HC (Table 2), while 2 proteins (CA125 and CRP) showed significant differences between RM and active cancer (Figure 1, Table S2 in File S1). The mean level of CA125 in RM samples is significantly reduced and similar to the value in HC, while the RC group has the highest mean CA125 (Table 2 and Table S2 in File S1). These results further validate CA125 as a good marker for monitoring ovarian cancer. The levels for CRP were also significantly reduced in RM samples as compared to active cancer and returned to almost normal levels (Table S2 in File S1).

**Figure 3:**
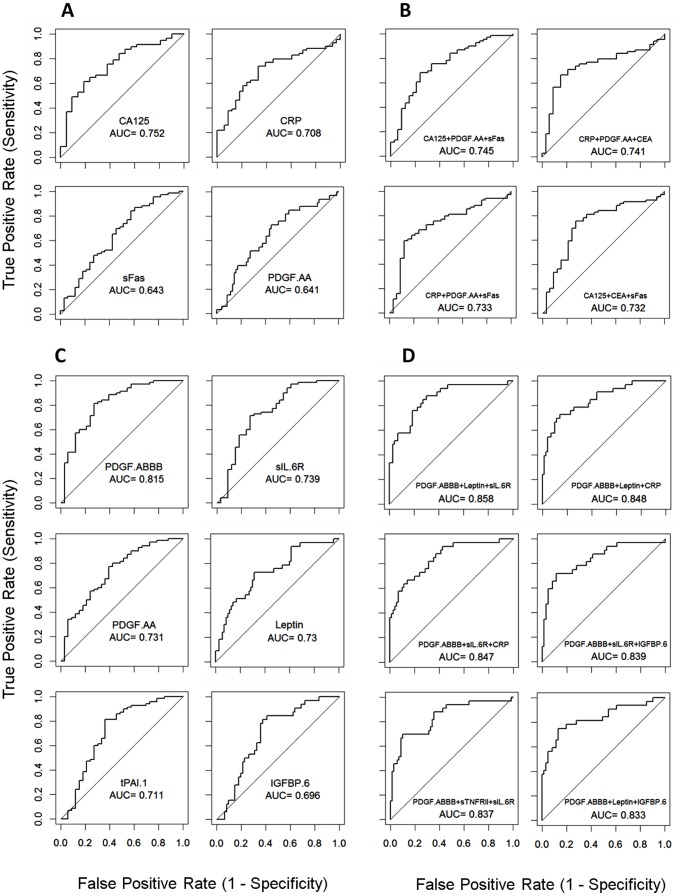
The ROC curves for the top molecules that distinguish samples at remission from samples with active cancer (A, B) or healthy controls (C, D). Results were shown for single proteins (A, C) and multi-marker models (B, D).

ROC analysis was also performed to identify individual molecules and 3-protein models that can best distinguish RM samples from cancer patients (PD+RC) or HC. The two best performing molecules that can distinguish RM from cancer are CA125 (AUC = 0.752) and CRP (AUC = 0.708) (Figure 3A), while the best molecules which can separate RM and HC are PDGF-AB/BB, PDGF-AA, sIL6R and Leptin (AUC = 0.815, 0.731, 0.739 and 0.73, respectively, Figure 3C). For 3-protein models we used the top 8 proteins and tested a total of 56 models. Protein combination models could not improve the AUC to distinguish RM samples from cancer patients (Figure 3B). However, to distinguish RM samples from HC, 2 models could achieve an AUC of more than 0.86 (Figure 3D) and 16 models could achieve an AUC of more than the best performing individual molecule.

### Serum Protein Profile at the PD Stage has Limited Prognostic Value

**Figure 4:**
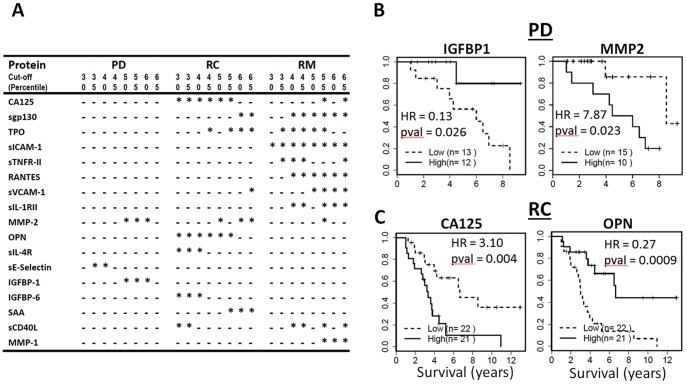
Survival analyses of ovarian cancer patients. Kaplan-Meier analysis was used to investigate the relationship of individual protein levels on overall survival in three different phenotypic groups (PD, RC, and RM). **A:** The subjects were assigned to the low or high expression groups based on the protein expression for each protein. As the best cutoff points were not known, we systematically evaluated eight cut-off points ranging from 30th percentile to 65th percentile of expression values. Each star (*) represents a significant difference in the overall survival of low expression and high expression groups. **B:** Survival curves of the samples from the PD stage. Only four proteins showed marginally significant associations with survival.

The impact of individual protein levels on survival was assessed using Kaplan-Meier analysis of 75 patients with survival data. The patients were assigned to the low or high expression groups based on the protein expression for each protein. As the best cutoff points were not known, we systematically evaluated eight cut-off points ranging from 30th percentile to 65th percentile of expression values. After the patients are assigned to one or the other group, log rank test was used to determine survival differences between the two groups. Survival analyses were performed separately for the PD, RC and RM samples. Using PD samples, only four proteins showed marginally significant associations with survival (Figure 4). We then evaluated the prognostic value of multivariate models that contain 4 proteins. For this purpose, k-means was used to cluster the patients into two groups based on the protein levels and Kaplan-Meier analyses were used to determine survival differences between the two clusters. Unfortunately, the multivariate models did not significantly improve the prognostic value of serum proteins measured at the PD stage.

### Five Serum Proteins at RM Stage Accurately Predict Therapeutic Outcomes

**Figure 5:**
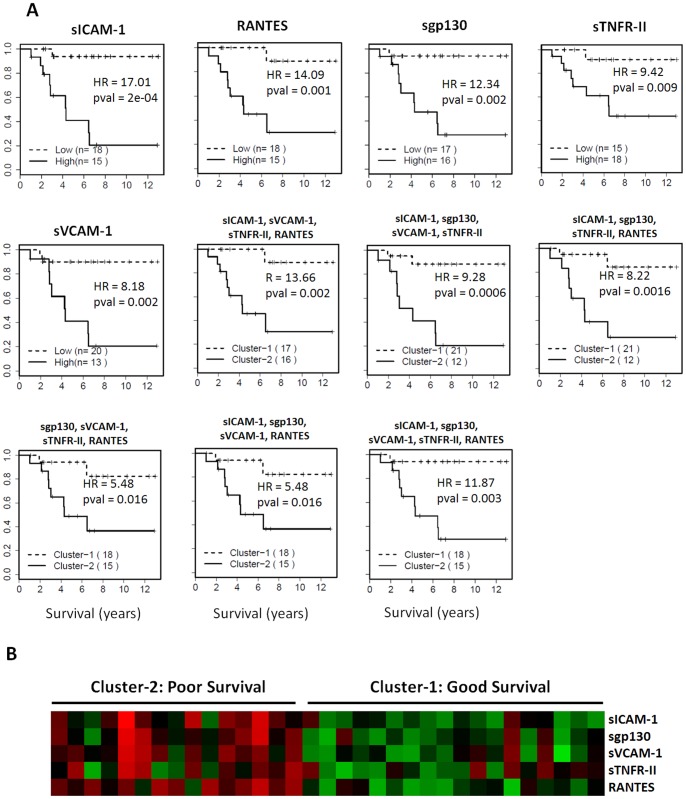
Survival analyses of the samples from the RM stage. **A:** The survival curves for the top five molecules that can distinguish patient subset with poor overall survival from patients with better survival. The prognostic value of multivariate models (combinations of 4 or 5 proteins) was determined by clustering the patients into two groups based on the expression levels of protein panels and survival differences were then determined between these two clusters using Kaplan-Meier analyses. **B:** The heatmap of protein expression in the samples from the RM stage. The patients with poor survival have higher expression levels for the five proteins.

Multiple proteins (sICAM1, RANTES, sgp130, sTNFR-II, sVCAM-1, CA125, TPO, MMP-2, sIL-1RII, sCD40L, and MMP-1) measured at the RM stage can individually predict overall survival of serous OC patients (Figure 4A). Among these proteins, five (sICAM1, RANTES, sgp130, sTNFR-II, sVCAM1) could separate the RM patients into two subgroups with distinct prognosis and sICAM-1 had the best prognostic value (HR = 17.01, p = 2×10^−4^, Figure 5). We also evaluated the prognostic value of all 5 models using 4 of the 5 proteins and the 5-protein model (Figure 5A). All six multi-marker models have excellent prognostic potential (HR = 5.48 to 13.66). Interestingly, the heatmap of protein expression (Figure 5B) clearly shows that the patients with poor survival have higher expression levels for the five proteins. Furthermore, examination of the distribution of other clinical parameters such as tumor grade and stage in the subgroups of patients with excellent versus poor survival indicate that the survival difference cannot be attributed to the known risk factors examined in this study.

## Discussion

The vast majority of the OC biomarker studies have focused on discovery and validation of biomarkers for diagnosis or early detection. CA125 is the best known OC biomarker and has been widely used in clinics, while HE4 was recently approved by the FDA as an OC biomarker. Despite the clinical value of CA125 and HE4, their utility in OC diagnosis, especially early detection, is quite limited due to inadequate specificity and sensitivity. A number of new OC biomarkers have been discovered in the last few years. Whereas the performance of these new biomarkers usually does not reach the level of CA125 or HE4, the combination of multiple biomarkers can improve the performance of CA125 [18]–[30]. Despite these advances, the currently available biomarkers may not have sufficient PPV suitable for population screening. Therefore, continuous effort in finding additional biomarkers is critical to improve patient care. In this study, we compared serum levels of 40 serum proteins and identified 25 proteins that were altered in OC compared to HC (Table 2). CRP and SAA proteins are highly increased, suggesting active inflammation in the patients with active disease (PD and RC) but to a lesser degree at the remission stage. Inflammation in OC is also indicated by the increased levels of soluble receptors such as sTNFR-II, sFas and sCD40L. Many of these molecules (CRP, SAA, sTNFR-II, IGFBP-2, Leptin, CD40L and sFas) have previously been reported in OC [22], [31]–[40]. The most down-regulated proteins are PDGF-AB/BB and PDGF-AA, two related molecules which play a critical role in cell proliferation and angiogenesis. Genomic studies suggested that activation of the PDGF pathway plays a critical role in OC [41]. While the pro-angiogenic and pro-growth function of PDGF would predict higher levels of serum PDGF [42], these two proteins are surprisingly lower in OC patients compared to HC. Consistent with our results, PDGF-AA was also reported to be significantly lower in sera of pancreatic cancer patients [43]. However, the implication of these observations remains to be elucidated.

The value of these serum proteins as OC biomarkers was evaluated using AUC from the ROC curves (Figure 2). PDGF-AB/BB, CA125, PDGF-AA and CRP had the highest AUC values (Figure 2). The AUC for CA125 was 0.847, which is comparable to many previous observations. Multivariate analysis was also carried out to evaluate the utility of using protein combinations as biomarkers. In many studies, researchers attempt to discover models with the best specificity and sensitivity using a large number of markers. While models using large numbers of proteins may perform better in the discovery dataset, they are generally more difficult to validate due to potential overfitting. Therefore, we evaluated models using small numbers of proteins. The AUC for 10 models with 3 proteins improved to 0.907–0.933 (Figure 2). These protein combinations are potentially useful for OC diagnosis and should be further evaluated for early OC detection in future studies.

Assessing therapeutic outcome and prognosis are of pivotal importance to the patients and their disease management. Numerous studies have evaluated and confirmed the value of CA125, leading to the mandatory test of CA125 for OC patients. Although normalization of CA125 is an important indication for remission, CA125 has serious limitations. First, a good proportion of OC patients have normal or near normal CA125 levels before chemotherapy. Second, it remains controversial whether CA125, tested either pre-operatively or post-operatively, has significant prognostic value. Several studies concluded that CA125 has no prognostic value [44], [45], while other studies reached the opposite conclusion [46]–[48]. Careful examination of these studies suggests that the controversy may be accounted for by small sample sizes of some studies and the low prognostic value of CA125. A recent study with a large sample size demonstrated a significant but relatively low prognostic value for CA125 (HR = 1.5) [49]. A number of studies also evaluated the prognostic value of other biomarkers or biomarker panels [50], [51]. Generally, these biomarkers have low HR when tested before chemotherapy. In this study, we also evaluated the prognostic value of the serum proteins that are significantly altered in serous OC samples post-diagnosis. Consistent with the previous findings, we were unable to identify individual proteins or combinations of proteins with appreciable value for prognosis when they are tested post-diagnosis (Figure 4).

In contrast, a number of proteins, when tested after therapy and at remission, have individually excellent prognostic value for overall survival of serous OC patients. The top five proteins (sICAM1, RANTES, sgp130, sTNFR-II and sVCAM1) have excellent HR (17.01, 14.09, 12.34, 9.42, and 8.18, respectively). All five possible combinations of 4 of the 5 proteins had excellent prognostic value (HR = 13.66, 9.28, 8.22, 5.48, 5.48). When all five proteins are used, the prognostic value was significant with HR = 11.87 (p = 0.003). The levels of these proteins are significantly higher in the patient subset with poor overall survival than in patients with better survival. Indeed, the RM samples from patients with poor survival had expression profiles indistinguishable from PD or RC samples, suggesting that the therapy was not completely successful for these patients. To our knowledge, these five serum proteins are the first reported panel that can accurately assess the therapeutic outcomes for therapy in serous OC patients; however, the modest sample size is the limitation of our study. The five proteins include two soluble adhesion molecules (sICAM1 and sVCAM1) and two soluble cytokine receptors (sTNFR-II and sgp130) critical to immune function. These results suggest that inflammation in response to residual tumor cells is evident in patients with poor survival even though the tumor load may be very low as indicated by a normal tumor antigen such as CA125. If our results are validated in longitudinal studies using a larger sample size, these biomarkers can be used together with CA125 and/or HE4, to more accurately assess the therapeutic outcome for OC patients.

## Supporting Information

File S1 **Tables S1–S3. Table S1:** Changes in serum protein levels in patients as compared to healthy controls. (PD: Post Diagnosis, HC: Healthy Controls, RC: Recurrence, RM: Remission). **Table S2:** Changes in serum protein levels in active cancer patients as compared to Remission cases. **Table S3:** AUC values of 56 Models (03 molecules in each model). Multivariate analysis was performed for classification of healthy controls and patients (HC vs PD+RC).
(DOCX)
