# Supplementary material for: Serum Protein Profile at Remission Can Accurately Assess Therapeutic Outcomes and Survival for Serous Ovarian Cancer
Source: PLoS One. 2013 Nov 11;8(11):e78393. doi: 10.1371/journal.pone.0078393 (PMC3823861; doi:10.1371/journal.pone.0078393)
Supplement: File S1 — Tables S1–S3. Table S1: Changes in serum protein levels in patients as compared to healthy controls. (PD: Post Diagnosis, HC: Healthy Controls, RC: Recurrence, RM: Remission). Table S2: Changes in serum protein levels in active cancer patients as compared to Remission cases. Table S3: AUC values of 56 Models (03 molecules in each model). Multivariate analysis was performed for classification of healthy controls and patients (HC vs PD+RC). (DOCX) [file pone.0078393.s001.docx]

**Table S1: Changes in serum protein levels in patients as compared to healthy controls (PD: Post Diagnosis, HC: Healthy Controls, RC: Recurrence, RM: Remission). The p-values are adjusted for multiple testing using FDR method.**

| **Protein** | **PD/HC** | **p-val** |  | **RC/HC** | **p-val** |  | **RM/HC** | **p-val** |
| --- | --- | --- | --- | --- | --- | --- | --- | --- |
| CA125 | **3.46** | **0.011** |  | **11.40** | **2E-07** |  | 1.56 | 0.249 |
| CRP | **3.52** | **0.037** |  | **6.35** | **2E-07** |  | **1.95** | **0.045** |
| PDGF-AB/BB | **0.39** | **1E-05** |  | **0.43** | **5E-07** |  | **0.52** | **2E-04** |
| PDGF-AA | **0.60** | **4E-04** |  | **0.56** | **1E-06** |  | **0.72** | **0.005** |
| sCD40L | **2.48** | **1E-04** |  | **2.26** | **7E-04** |  | **2.27** | **3E-04** |
| IGFBP-2 | **4.69** | **2E-04** |  | 1.63 | 0.279 |  | 1.27 | 0.603 |
| sFas | **1.57** | **2E-04** |  | **1.33** | **0.006** |  | 1.13 | 0.322 |
| sIL-6R | **0.74** | **0.008** |  | **0.74** | **2E-04** |  | **0.75** | **0.002** |
| SAA | 2.81 | 0.080 |  | **4.11** | **3E-04** |  | 1.57 | 0.230 |
| Leptin | 1.13 | 0.796 |  | **2.91** | **4E-04** |  | **2.79** | **0.001** |
| sVCAM-1 | 0.86 | 0.185 |  | **0.75** | **7E-04** |  | 0.84 | 0.052 |
| MDC | **0.69** | **0.004** |  | **0.72** | **0.020** |  | **0.79** | **0.042** |
| sIL-4R | 0.90 | 0.549 |  | **0.68** | **0.004** |  | 0.82 | 0.220 |
| sE-SELECTIN | **0.66** | **0.011** |  | **0.71** | **0.006** |  | **0.71** | **0.039** |
| tPAI-1 | **0.73** | **0.018** |  | **0.74** | **0.011** |  | **0.73** | **0.006** |
| CD14 | **1.29** | **0.006** |  | **1.22** | **0.042** |  | 1.11 | 0.309 |
| MMP-1 | 1.67 | 0.097 |  | **1.98** | **0.008** |  | 1.21 | 0.490 |
| sTNFRII | **1.45** | **0.015** |  | **1.31** | **0.014** |  | 1.17 | 0.180 |
| IGFBP-6 | **1.56** | **0.015** |  | **1.40** | **0.048** |  | 1.35 | 0.093 |
| HGF | 1.21 | 0.263 |  | **1.31** | **0.018** |  | 1.19 | 0.309 |
| sICAM-1 | 0.87 | 0.309 |  | 0.86 | 0.222 |  | **0.78** | **0.024** |
| IGFBP-1 | **1.81** | **0.025** |  | 0.99 | 0.974 |  | 1.20 | 0.546 |
| sIL-2Ra | **1.38** | **0.038** |  | 1.08 | 0.616 |  | 0.97 | 0.853 |
| MMP-9 | 1.39 | 0.309 |  | 1.55 | 0.053 |  | **1.55** | **0.045** |
| CA15-3 | 1.33 | 0.263 |  | **1.56** | **0.049** |  | 1.17 | 0.501 |
| sgp130 | 0.89 | 0.309 |  | 0.84 | 0.052 |  | 0.85 | 0.093 |
| CEA | 0.85 | 0.544 |  | 1.23 | 0.376 |  | 0.71 | 0.080 |
| RANTES | 0.71 | 0.087 |  | 0.82 | 0.309 |  | 0.71 | 0.080 |
| MIG | 0.74 | 0.257 |  | 0.69 | 0.136 |  | 0.65 | 0.093 |
| MMP-2 | 1.01 | 0.954 |  | 0.84 | 0.108 |  | 0.86 | 0.198 |
| sIL-1RII | 1.16 | 0.221 |  | 1.04 | 0.742 |  | 0.87 | 0.546 |
| OPG | 1.23 | 0.309 |  | 1.16 | 0.413 |  | 0.94 | 0.840 |
| MCP-1 | 1.15 | 0.337 |  | 0.90 | 0.513 |  | 1.02 | 0.919 |
| PTH | 1.35 | 0.400 |  | 1.16 | 0.653 |  | 1.04 | 0.929 |
| TPO | 1.33 | 0.415 |  | 1.22 | 0.529 |  | 1.16 | 0.677 |
| GRO | 0.93 | 0.677 |  | 1.08 | 0.639 |  | 0.87 | 0.444 |
| IGFBP-3 | 0.93 | 0.603 |  | 1.08 | 0.546 |  | 0.89 | 0.513 |
| OPN | 1.12 | 0.662 |  | 1.09 | 0.750 |  | 0.85 | 0.552 |
| IGFBP-7 | 1.10 | 0.569 |  | 1.09 | 0.569 |  | 1.05 | 0.747 |
| sEGFR | 1.02 | 0.867 |  | 0.99 | 0.957 |  | 1.04 | 0.779 |

**Table S2: Changes in serum protein levels in active cancer patients as compared to Remission cases (PD: Post Diagnosis, RC: Recurrence, RM: Remission). The p-values are adjusted for multiple testing using FDR method.**

| **Protein** | **(PD+RC)/RM** | **adj-pval** |
| --- | --- | --- |
| CA125 | 4.74 | 2E-03 |
| CRP | 2.63 | 0.03 |
| SAA | 2.29 | 0.16 |
| CEA | 1.51 | 0.21 |
| sFas | 1.24 | 0.21 |
| PDGF.AA | 0.79 | 0.22 |
| MMP.1 | 1.53 | 0.41 |
| IGFBP.2 | 1.89 | 0.50 |
| OPN | 1.29 | 0.50 |
| sTNFRII | 1.17 | 0.50 |
| PDGF.ABBB | 0.80 | 0.53 |
| CD14 | 1.12 | 0.56 |
| CA15.3 | 1.26 | 0.56 |
| sICAM.1 | 1.12 | 0.56 |
| sIL.1RII | 1.24 | 0.56 |
| sIL.2Ra | 1.22 | 0.56 |
| IGFBP3 | 1.14 | 0.57 |
| Leptin | 0.74 | 0.57 |
| MDC | 0.90 | 0.57 |
| OPG | 1.25 | 0.57 |
| GRO | 1.18 | 0.60 |
| sVCAM.1 | 0.94 | 0.72 |
| IGFBP.6 | 1.07 | 0.77 |
| PTH | 1.18 | 0.89 |
| RANTES | 1.09 | 0.89 |
| sIL.4R | 0.92 | 0.89 |
| HGF | 1.07 | 0.90 |
| MIG | 1.09 | 0.90 |
| MMP.2 | 1.04 | 0.90 |
| IGFBP.7 | 1.04 | 0.92 |
| MCP.1 | 0.97 | 0.92 |
| MMP.9 | 0.96 | 0.92 |
| sCD40L | 1.03 | 0.92 |
| sEGFR | 0.96 | 0.92 |
| sE.SELECTIN | 0.97 | 0.92 |
| TPO | 1.08 | 0.92 |
| IGFBP.1 | 1.03 | 0.96 |
| sIL.6R | 0.99 | 0.97 |
| sgp130 | 1.00 | 0.99 |
| tPAI.1 | 1.00 | 0.99 |

**Table S3: AUC values of 56 Models (03 molecules in each model). Multivariate analysis was performed for classification of healthy controls and patients (HC vs PD+RC).**

| SNo. | | Mol1 | | Mol2 | | Mol3 | | AUC | | |
| --- | --- | --- | --- | --- | --- | --- | --- | --- | --- | --- |
| 1 | PDGF-ABBB | | sFas | | CA125 | | 0.933 | |  |  |
| 2 | PDGF-ABBB | | CRP | | CA125 | | 0.929 | |  |  |
| 3 | PDGF-ABBB | | CA125 | | sTNFRII | | 0.928 | | |  |
| 4 | PDGF-ABBB | | CA125 | | SAA | | 0.922 | | |  |
| 5 | PDGF-ABBB | | PDGF-AA | | CA125 | | 0.918 | | |  |
| 6 | PDGF-ABBB | | CA125 | | IGFBP-6 | | 0.917 | | |  |
| 7 | PDGF-ABBB | | CRP | | sFas | | 0.915 | | |  |
| 8 | PDGF-ABBB | | PDGF-AA | | CRP | | 0.907 | | |  |
| 9 | PDGF-ABBB | | CRP | | IGFBP-6 | | 0.907 | | |  |
| 10 | PDGF-AA | | CA125 | | sTNFRII | | 0.907 | | |  |
| 11 | PDGF-ABBB | | CRP | | SAA | | 0.906 | | |  |
| 12 | PDGF-ABBB | | CRP | | sTNFRII | | 0.903 | | |  |
| 13 | PDGF-AA | | sFas | | CA125 | | 0.900 | | |  |
| 14 | PDGF-AA | | CRP | | CA125 | | 0.899 | | |  |
| 15 | PDGF-AA | | CA125 | | IGFBP-6 | | 0.893 | | |  |
| 16 | PDGF-AA | | CRP | | sFas | | 0.891 | | |  |
| 17 | PDGF-AA | | CA125 | | SAA | | 0.89 | | |  |
| 18 | PDGF-AA | | CRP | | IGFBP-6 | | 0.889 | | |  |
| 19 | PDGF-ABBB | | sFas | | SAA | | 0.88 | | |  |
| 20 | PDGF-ABBB | | SAA | | sTNFRII | | 0.88 | | |  |
| 21 | PDGF-AA | | CRP | | sTNFRII | | 0.88 | | |  |
| 22 | PDGF-AA | | CRP | | SAA | | 0.876 | | |  |
| 23 | PDGF-ABBB | | sFas | | sTNFRII | | 0.875 | | |  |
| 24 | PDGF-ABBB | | SAA | | IGFBP-6 | | 0.875 | | |  |
| 25 | PDGF-ABBB | | PDGF-AA | | SAA | | 0.872 | | |  |
| 26 | PDGF-ABBB | | PDGF-AA | | sTNFRII | | 0.872 | | |  |
| 27 | PDGF-ABBB | | PDGF-AA | | sFas | | 0.867 | | |  |
| 28 | PDGF-ABBB | | sFas | | IGFBP-6 | | 0.863 | | |  |
| 29 | PDGF-AA | | SAA | | sTNFRII | | 0.863 | | |  |
| 30 | PDGF-AA | | sFas | | SAA | | 0.859 | | |  |
| 31 | PDGF-AA | | sFas | | sTNFRII | | 0.858 | | |  |
| 32 | PDGF-ABBB | | sTNFRII | | IGFBP-6 | | 0.857 | | |  |
| 33 | PDGF-AA | | SAA | | IGFBP-6 | | 0.857 | | |  |
| 34 | PDGF-ABBB | | PDGF-AA | | IGFBP-6 | | 0.854 | | |  |
| 35 | PDGF-AA | | sFas | | IGFBP-6 | | 0.854 | | |  |
| 36 | PDGF-AA | | sTNFRII | | IGFBP-6 | | 0.847 | | |  |
| 37 | CRP | | sFas | | CA125 | | 0.84 | | |  |
| 38 | sFas | | CA125 | | sTNFRII | | 0.832 | | |  |
| 39 | sFas | | CA125 | | SAA | | 0.827 | | |  |
| 40 | CRP | | CA125 | | IGFBP-6 | | 0.825 | | |  |
| 41 | CRP | | CA125 | | sTNFRII | | 0.823 | | |  |
| 42 | sFas | | CA125 | | IGFBP-6 | | 0.822 | | |  |
| 43 | CA125 | | SAA | | sTNFRII | | 0.812 | | |  |
| 44 | CA125 | | SAA | | IGFBP-6 | | 0.81 | | |  |
| 45 | CRP | | sFas | | IGFBP-6 | | 0.806 | | |  |
| 46 | CRP | | CA125 | | SAA | | 0.804 | | |  |
| 47 | CRP | | sFas | | SAA | | 0.797 | | |  |
| 48 | CA125 | | sTNFRII | | IGFBP-6 | | 0.796 | | |  |
| 49 | CRP | | sFas | | sTNFRII | | 0.794 | | |  |
| 50 | CRP | | sTNFRII | | IGFBP-6 | | 0.784 | | |  |
| 51 | CRP | | SAA | | IGFBP-6 | | 0.783 | | |  |
| 52 | CRP | | SAA | | sTNFRII | | 0.775 | | |  |
| 53 | sFas | | SAA | | IGFBP-6 | | 0.746 | | |  |
| 54 | sFas | | SAA | | sTNFRII | | 0.743 | | |  |
| 55 | SAA | | sTNFRII | | IGFBP-6 | | 0.722 | | |  |
| 56 | sFas | | sTNFRII | | IGFBP-6 | | 0.717 | | |  |
